# Theoretical Relationship Between Two Measures of Spike Synchrony: Correlation Index and Vector Strength

**DOI:** 10.3389/fnins.2021.761826

**Published:** 2021-12-20

**Authors:** Dominik Kessler, Catherine E. Carr, Jutta Kretzberg, Go Ashida

**Affiliations:** ^1^Computational Neuroscience, Department of Neuroscience, Faculty VI, University of Oldenburg, Oldenburg, Germany; ^2^Department of Biology, University of Maryland, College Park, MD, United States; ^3^Cluster of Excellence Hearing4all, Department of Neuroscience, Faculty VI, University of Oldenburg, Oldenburg, Germany

**Keywords:** phase-locking, circular statistics, temporal coding, auditory brainstem, autocorrelogram, spike train analysis, periodic signals, frequency-following response

## Abstract

Information processing in the nervous system critically relies on temporally precise spiking activity. In the auditory system, various degrees of phase-locking can be observed from the auditory nerve to cortical neurons. The classical metric for quantifying phase-locking is the vector strength (VS), which captures the periodicity in neuronal spiking. More recently, another metric, called the correlation index (CI), was proposed to quantify the temporally reproducible response characteristics of a neuron. The CI is defined as the peak value of a normalized shuffled autocorrelogram (SAC). Both VS and CI have been used to investigate how temporal information is processed and propagated along the auditory pathways. While previous analyses of physiological data in cats suggested covariation of these two metrics, general characterization of their connection has never been performed. In the present study, we derive a rigorous relationship between VS and CI. To model phase-locking, we assume Poissonian spike trains with a temporally changing intensity function following a *von Mises* distribution. We demonstrate that VS and CI are mutually related via the so-called concentration parameter that determines the degree of phase-locking. We confirm that these theoretical results are largely consistent with physiological data recorded in the auditory brainstem of various animals. In addition, we generate artificial phase-locked spike sequences, for which recording and analysis parameters can be systematically manipulated. Our analysis results suggest that mismatches between empirical data and the theoretical prediction can often be explained with deviations from the *von Mises* distribution, including skewed or multimodal period histograms. Furthermore, temporal relations of spike trains across trials can contribute to higher CI values than predicted mathematically based on the VS. We find that, for most applications, a SAC bin width of 50 ms seems to be a favorable choice, leading to an estimated error below 2.5% for physiologically plausible conditions. Overall, our results provide general relations between the two measures of phase-locking and will aid future analyses of different physiological datasets that are characterized with these metrics.

## 1. Introduction

Temporal coding is found virtually everywhere in the brain, underlying numerous sensory, cognitive, and motor functions of the nervous system (Paton and Buonomano, 2018). Well-timed neuronal spiking is essential for vision (Rucci et al., 2018), audition (Grothe et al., 2010; Heil and Peterson, 2015; Yin et al., 2019), balance (Cullen, 2012), olfaction (Gire et al., 2013), gustation (Hallock and Di Lorenzo, 2006), touch (Saal et al., 2016), electrosensation (Carr and Friedman, 1999; Baker et al., 2013), as well as spinal pattern generation (Catela et al., 2015), motor control (Lehman and Bartussek, 2017; Sober et al., 2018), cerebellar motor learning (De Zeeuw et al., 2011), and memory formation and consolidation (Dragoi, 2020). Loss of temporal precision in neural activity is related to cognitive and behavioral deficits (Balci et al., 2009). When action potentials of a neuron occur preferentially at a certain phase of a periodic stimulus, such a neuronal spiking pattern is called “phase-locked” (see Carr and Friedman, 1999; Ashida et al., 2010, for typical examples). In the auditory system, phase-locking to tonal stimuli plays a fundamental role, for example, in detecting the location of a sound source (Grothe et al., 2010; Ashida, 2015; Yin et al., 2019).

**Figure 1:**
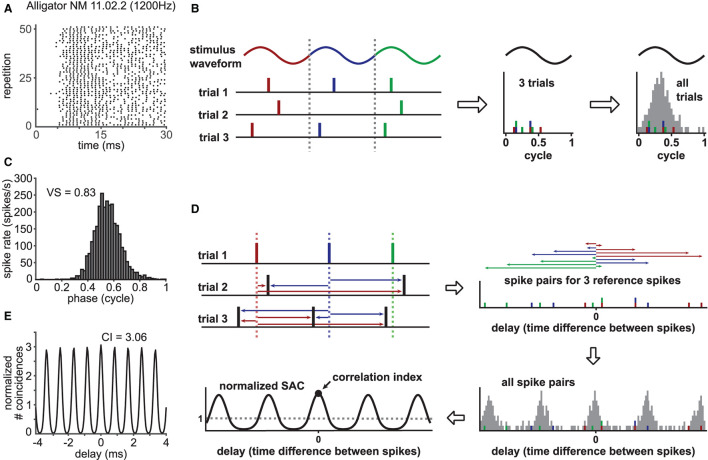
Characterization of phase-locked spiking response. **(A)** Exemplary phase-locked spike trains recorded in a nucleus magnocellularis (NM) neuron of an American alligator. The raster plot shows the occurrence of spikes for 51 (out of 63) repetitions of a 100 ms pure tone stimulus at the unit's CF (1200 Hz). Sound stimulation starts at 0 ms. **(B)** Schematic drawing for the construction of a phase histogram. For each recorded spike, the corresponding phase at the reference frequency is calculated. Then, a histogram is constructed to show the number of spikes for each phase bin. A steep peak indicates strong phase-locking, whereas a flat phase histogram reflects a lack thereof. **(C)** Phase histogram for the response in **(A)** with its vector strength (VS) value. **(D)** Schematic drawing for the construction of a normalized shuffled autocorrelogram (SAC). Each spike timing within one spike train (here, exemplary trial 1) is compared to the spike timings of all other trains (here, trial 2 and 3) by measuring pairwise temporal distances of the spikes (colored arrows). This procedure is repeated for every recorded spike train. All resulting temporal distances, also called delays, are binned into a histogram and normalized to a dimensionless entity with an average of one. The correlation index (CI) corresponds to the value of the SAC at delay zero. **(E)** SAC for the response in **(A)** with its CI value.

Phase-locking of a spike train (Figure 1A) can be captured in a phase histogram (Figure 1C) that visualizes the spiking rate at each phase of the periodic reference signal (see Figure 1B for the schematic construction of a phase histogram). In order to quantify the degree of phase-locking, a metric called the “vector strength” (VS: Goldberg and Brown, 1969; Fisher, 1993; van Hemmen, 2013) has been widely used (for examples, see Joris et al., 2004; Ashida et al., 2010; Heil and Peterson, 2015, 2017). Here, each spike is converted into a vector on a unit circle with a corresponding phase; all these phase vectors are summed up to a mean spike vector, whose length is the value of VS. Mathematically, VS (sometimes called “synchronization index” or “synchronization coefficient”) is equal to the absolute value of the Fourier component of the spike train at the reference signal frequency (*f*) normalized by the total number of spikes (Johnson, 1974; Ashida et al., 2010). By definition (detailed in Materials and Methods), the value of VS is between zero and one: from no to perfect periodicity at *f*. Certain types of neurons in the cochlear nucleus, for instance, exhibit enhanced phase-locking compared to their peripheral auditory nerve inputs, which is manifested in an increase of VS (Joris et al., 1994; Köppl, 1997; Wei et al., 2017).

In addition to VS, another metric called the “correlation index” (CI: Joris et al., 2006) was more recently introduced to quantify neuronal synchrony. The value of CI is defined as the height of the central peak of a normalized shuffled autocorrelogram (SAC; Figure 1E), whose construction is depicted in Figure 1D (a more detailed description is given in Materials and Methods). If a set of spike trains has no temporally reproducible spiking pattern across trials, its CI value equals one. A large CI value indicates highly reproducible spiking responses over repeated trials (Joris et al., 2006). In contrast to VS, which is narrowband (i.e., only the spectral component at frequency *f* is considered), CI is generally broadband, as it is a collective measure of multiple frequency components (Parida et al., 2021). SACs and CIs can be applied to both periodic and aperiodic signals and were used for characterizing the temporal coding properties of auditory nerve (AN) fibers (Dreyer and Delgutte, 2006; Heinz and Swaminathan, 2009; Huet et al., 2018; Heeringa et al., 2019), various neurons in the cochlear nuclei recorded *in vivo* (Gai and Carney, 2008; Steinberg and Peña, 2011; Recio-Spinoso, 2012; Keine et al., 2016) and *in vitro* (Street and Manis, 2007; Kreeger et al., 2012), and neurons in the auditory midbrain (Shackleton et al., 2009; Zheng and Escabí, 2013).

While both VS and CI represent the degree of synchronized neuronal spiking activity, it is not known how these two measures are related to each other. Previous analyses of physiological data suggested that they should covary according to some monotonic, non-linear relationship (Dreyer and Delgutte, 2006; Joris et al., 2006). Parida and colleagues mathematically related peristimulus time histograms (PSTHs) to VS and CI under very general conditions (Parida et al., 2021). In the present study, we derive a mathematically rigorous relationship between VS and CI under certain assumptions for phase-locking. Our theoretical results will be validated with spike train data that were either recorded from different types of auditory neurons or simulated with available auditory neuron models. These results provide a foundation for comparing past and future experimental data quantified with these measures of phase-locking.

## 2. Materials and Methods

In this section, we summarize our theoretical formulations and describe the methods for validating our mathematical results. A full derivation of the equations is provided in Supplementary Materials.

### 2.1. Mathematical Analysis

#### 2.1.1. *Von Mises* Distribution

**Figure 2:**
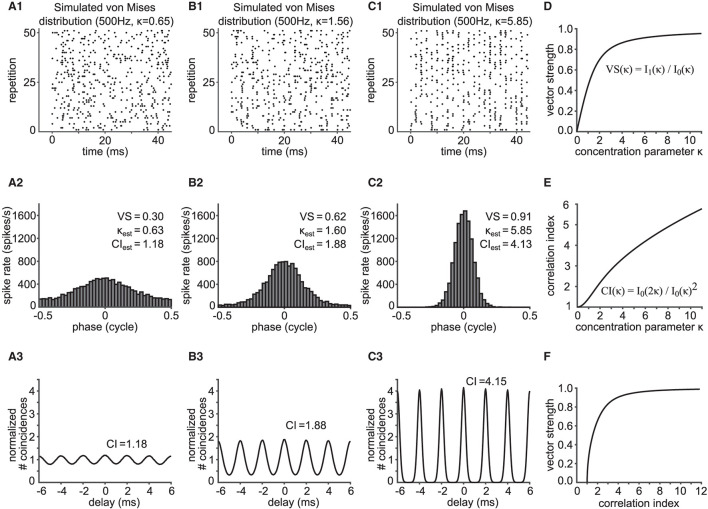
Relationship between vector strength (VS) and correlation index (CI). **(A1,B1,C1)** Phase-locked spike trains simulated with an inhomogeneous Poisson process whose periodic intensity function is *von Mises* distributed. The raster plots show the spike occurrences for three different phase-locking strengths, namely κ = 0.65 **(A1)**, κ = 1.56 **(B1)** and κ = 5.85 **(C1)**. **(A2,B2,C2)** Corresponding phase histograms for the simulated spike trains in **(A1–C1)**. The resulting empirical VS is shown, together with its back-calculated κ and estimated CI value. **(A3,B3,C3)** SACs for the spike trains in **(A1–C1)** with corresponding empirical CI values. In **(A1–C1)**, only 51 trials are shown for clarity, while 400 trials of 150 ms trains were used for **(A2–C2)** and **(A3–C3)**. **(D)** Value of VS as a function of κ for the *von Mises* distribution. **(E)** Value of CI as a function of κ for the *von Mises* distribution. **(F)** Relation between VS and CI for varied concentration parameters κ of the underlying *von Mises* distribution.

A key assumption for our mathematical formulation and the modeling of phase-locked spike trains is that the neuron fires action potentials according to an underlying *von Mises* distribution (Fisher, 1993). Such an assumption has been adopted in previous modeling studies of auditory neurons for half a century (e.g., Siebert, 1970; Colburn, 1973; Johnson, 1974; Ashida and Carr, 2010). A more recent study confirmed that most phase histograms of cat auditory nerve fibers can be reasonably described by the *von Mises* distribution (Peterson and Heil, 2020). Here, we consider a *von Mises* density function *p*_κ, *f*_, whose period is *T* = 1/*f*, with *f* being the stimulus (or reference) frequency, as


(1) $$\begin{array}{r} p_{\kappa ,f}\left(t\right)=\frac{1}{T\cdot I_{0}\left(\kappa \right)}\exp\;\kappa \cos\left(2\pi ft-\mu \right)\;, \end{array}$$

where μ is the mean direction (or the preferred spiking phase) and κ≥0 is the concentration parameter. Generally, a large κ corresponds to a high degree of phase-locking (numerical examples are shown in Figures 2A1–C3). $I_{0}\left(\kappa \right)=\frac{1}{2\pi }\int_{-\pi }^{\pi }\exp\left(\kappa \cos\left(x\right)\right)\text{d}x$ is the modified Bessel function of order zero, which serves as the normalization constant for *p*_κ, *f*_, such that $\int_{0}^{T}p_{\kappa ,f}\left(t\right)\text{d}t=1$. Without loss of generality, we set μ = 0 for the rest of this paper.

For generating phase-locked spike trains, we used an inhomogeneous Poisson process with a periodic time-varying intensity function


(2) $$\begin{array}{r} \lambda \left(t\right)=T\bar{\lambda }\cdot p_{\kappa ,f}\left(t\right), \end{array}$$

where *T* is the length of the stimulus period, and $\bar{\lambda }$ the mean spike rate of the neuron (see Ashida et al., 2010). In the following analyses, VS, SAC, and CI do not depend on $\bar{\lambda }$. We note that the presented results are asymptotically true with sufficiently many spikes collected. When only few spikes are considered, the results would be biased (Kutil, 2012). Furthermore, phase histograms of real neurons do not necessarily follow the *von Mises* distribution because of refractory periods and harmonic distortions (e.g., Avissar et al., 2013; Peterson and Heil, 2019). We will revisit this point in the Discussion.

#### 2.1.2. Vector Strength

As reviewed in Ashida et al. (2010), the vector strength of a neuronal spike train in response to a periodic signal with frequency *f* is calculated as the length of the mean phase vector (X, Y) of all collected spikes (Goldberg and Brown, 1969), namely $\text{VS}=\sqrt{{\text{X}}^{2}+{\text{Y}}^{2}}$ with $\text{X}=\frac{1}{N}\sum_{j=1}^{N}\sin\left(2\pi ft_{j}\right)$ and $\text{Y}=\frac{1}{N}\sum_{j=1}^{N}\cos\left(2\pi ft_{j}\right)$, where *t*_*j*_ denotes the timing of the *j*-th spike and *N* is the total number of spikes. For our theoretical analyses, we used the continuous extension of the definition of vector strength (see Ashida et al., 2010, 2013), according to which VS was computed as a function of κ by


(3) $$\begin{array}{r} \text{VS}\left(\kappa \right)=\frac{1}{T\cdot I_{0}\left(\kappa \right)}\int_{0}^{T}\exp\;\kappa \cos\left(2\pi ft\right)\;\cos\left(2\pi ft\right)\text{d}t=\frac{I_{1}\left(\kappa \right)}{I_{0}\left(\kappa \right)}, \end{array}$$

where $I_{1}\left(\kappa \right)=\frac{1}{2\pi }\int_{-\pi }^{\pi }\exp\left(\kappa \cos\left(x\right)\right)\cos\left(x\right)\text{d}x$ is the modified Bessel function of order one. VS monotonically increases with κ (Figure 2D).

#### 2.1.3. Normalized Shuffled Autocorrelogram and Correlation Index

SACs are obtained by measuring all pairwise spike intervals across all non-identical spike trains (see Figure 1D). The peak value of the normalized SAC at delay zero is the CI, which captures the degree of reproducibility in spike trains for repeated stimuli. Assuming that spike trains to repeated stimulus presentations are independent and identically *von Mises* distributed, we can derive a formula for SAC, which is only dependent on the concentration parameter κ and the time delay *s* (see Supplementary Material for detailed derivations). Namely,


(4) $$\begin{array}{l} {\text{SAC}}_{\kappa }\left(s\right)=T\cdot \int_{0}^{T}p_{\kappa ,f}\left(t\right)\cdot p_{\kappa ,f}\left(t-s\right)\text{d}t\\ =\frac{1}{T\cdot I_{0}{\left(\kappa \right)}^{2}}\int_{0}^{T}\exp\;\kappa \;\cos\left(2\pi ft\right)+\cos\left(2\pi f\left(t-s\right)\right)\;\;\text{d}t\\ =\frac{1}{I_{0}{\left(\kappa \right)}^{2}}\cdot \frac{1}{T}\int_{0}^{T}\exp\;2\kappa \cos\left(\pi fs\right)\cdot \cos\;2\pi f\;t-\frac{s}{2}\;\;\;\text{d}t\\ =\frac{I_{0}\left(2\kappa \cos\left(\pi fs\right)\right)}{I_{0}{\left(\kappa \right)}^{2}}. \end{array}$$

For its center peak at delay *s* = 0, we find


(5) $$\begin{array}{r} \text{CI}\left(\kappa \right)={\text{SAC}}_{\kappa }\left(0\right)=\frac{I_{0}\left(2\kappa \right)}{I_{0}{\left(\kappa \right)}^{2}}. \end{array}$$

CI increases monotonically as a function of κ (Figure 2E). Knowing the relationship between the concentration parameter κ with VS and CI, we can now relate VS and CI via κ (Figure 2F). We note that there is no closed formula that directly relates VS and CI without using κ. Therefore, to estimate the value of CI from the value of VS, for example, Equation (3) is solved for κ, and the obtained value of κ is then substituted into Equation (5).

#### 2.1.4. Effects of Data Length ***D*** and SAC Bin Width ***ω***

To evaluate the scattering of the data around the theoretical VS-CI curve, we examined the effect of the data length *D* and the bin width ω on the SAC and CI value. Here we refer to the “data length” as the time length of each single spike train used for the calculation of SAC. Assuming that it is equally likely for a unit to fire during each stimulus phase, the data length *D* introduces a linear decay to the side peaks of the SAC with delay *s*, which can be combined with the SAC in Equation (4) by the multiplicative factor


(6) $$\zeta_{D}(s)=\left\{ \begin{array}{l} 1-|\frac{s}{D}|,\;|s|<D\\ \;0\;,\;|s|\geq D \end{array}\right.,$$

such that


$$\begin{array}{l} {\text{SAC}}_{\kappa ,D}\left(s\right)={\text{SAC}}_{\kappa }\left(s\right)\cdot \zeta_{D}\left(s\right). \end{array}$$

This equation indicates that CI is not affected by the data length *D*, as it is the value of SAC at *s* = 0.

The derivation of the CI in Equation (5) implicitly assumed a sufficiently small ω. Therefore, by design, the value of the *von Mises* density *p*_κ, *f*_ does not change within each bin. This is different when introducing binning of a finite size, which leads to a bias in the calculation of CI. The effect of binning can be formulated as the convolution of *p*_κ, *f*_ with a rectangular window function. This effectively induces the averaging of function values in each bin. Thus, CI becomes a function of ω, κ and *f* (see Supplementary Material for detailed derivation):


(7) $$\begin{array}{r} {\text{CI}}_{\omega }\left(\kappa \right)=1+2\overset{\infty }{\underset{n=1}{\sum }}\underset{\begin{array}{c} \text{strength of}\\ \text{n-th harmonic} \end{array}}{\underset{︸}{\;\frac{I_{n}\left(\kappa \right)}{I_{0}\left(\kappa \right)}{\;}^{2}}}\underset{\begin{array}{c} \text{decay factor} \end{array}}{\underset{︸}{\;\frac{\sin\left(\pi nf\omega \right)}{\pi nf\omega }\;}}, \end{array}$$

For large bin widths ω, the CI value decays toward 1, as the second term of Equation (7) monotonically decreases with ω. For sufficiently small ω, the decay factor is (close to) 1, since sin(π*nfω*)≈π*nfω*, thus leading to very small bias.

### 2.2. Data Analysis

To validate our theoretical findings, we analyzed spike trains from three different sources: (1) simulated spike trains with an inhomogeneous Poisson process, (2) simulated auditory nerve (AN) and globular bushy cell (GBC) data, and (3) spike timing data recorded from auditory neurons *in vivo*. To calculate the SACs and CI values, custom-written Matlab scripts were used that followed the procedure described in Joris et al. (2006). Unless otherwise stated, we used ω = 50 μs for the SAC bin width.

#### 2.2.1. Spike Train Generation

Phase-locked spike trains were generated using an inhomogeneous Poisson process with the time-dependent intensity function in Equation (2). This simple model does not include refractory periods (see Discussion for the possible effects of refractory periods on VS and CI). A total of 46 units were generated with target VS values evenly spaced between 0.05 and 0.95 (with a step of 0.02), for which the value of κ was back-calculated using Equation (3). The frequency *f* was fixed to 500 Hz. For each κ, 150 ms long spike trains were generated 400 times. The average firing rate was set to 200 spikes/s and the time resolution for spike generation was 2 μs (i.e., ω/dt = 25). Figures 2A1–C1 shows exemplary rasters of simulated spike trains with target VS values of 0.31, 0.61 and 0.91. Their corresponding phase histograms (Figures 2A2–C2) and SACs (Figures 2A3–C3) are shown below the rasters.

#### 2.2.2. AN/GBC Model Data

To simulate AN fiber responses, an auditory periphery model (Bruce et al., 2018) was used, which was shown to replicate physiological spiking patterns of AN in cats. To simulate tone-driven spiking patterns of GBCs, an adaptive coincidence counting model was used, which was fed with the simulated AN output (Ashida et al., 2019). AN fiber spike trains were generated for tonal waveforms with frequencies ranging from 200 to 3,000 Hz in steps of 100 Hz at sound pressure levels of 40 and 70 dB SPL. The spontaneous spike rate of the AN model was set to 70 spikes/s, so that we could reuse the simulated spike trains of this high spontaneous rate AN fiber model as inputs to the GBC model. The characteristic frequency of the model was always the same as the stimulus frequency. The simulated time length of each trial was 190 ms and the sound stimulation started at 25 ms with a 5 ms cosine ramp. Sampling frequency was 100 kHz (dt = 10μs). For each combination of sound frequency and level, 8000 repetitions were generated. The first 400 trials were used for further AN data analyses. The entire 8000 repetitions served as inputs to simulated GBCs. Assuming that each GBC received inputs from 20 AN units (Ashida et al., 2019), we had 400 trials for each GBC simulation. For both AN and GBC, the resulting spike trains were analyzed over a 150 ms window, excluding the first 15 ms onset response to avoid possible effects of spike rate adaptation on VS and CI.

#### 2.2.3. *In vivo* Recordings From Auditory Brainstem Neurons

Similarly to Ashida and Carr (2010), we re-analyzed our previous *in vivo* recording data from nucleus magnocellularis (NM) and nucleus laminaris (NL) neurons in birds and reptiles. Corresponding experimental procedures were described in Carr et al. (2009) for alligators, in Köppl and Carr (2008) for chickens, and in Carr and Köppl (2004) for barn owls. To induce phase-locked spiking responses, NM and NL neurons in these animals were driven with repeated tonal stimulation at their best (or characteristic) frequencies. In total, we re-analyzed 142 units (57 from alligators, 34 from chickens, and 51 from owls). Stimulus duration was 50 or 100 ms, except for two units with 200 and 400 ms stimulation. Stimulus presentation was repeated 50–400 times. To eliminate the effect of the stimulus onset, we excluded the initial 15 ms of the spiking response for each stimulus presentation. Units with a total number of collected spikes fewer than 400 or with an average driven firing rate lower than 30 spikes/s were excluded from further analyses, leaving us with 125 units. From the spike timing data of each of these units, we calculated VS and CI according to the methods described above. In addition to our own data, we adopted the empirical VS-CI plot of Joris et al. (2006) for comparison, in which *in vivo* recording data from trapezoid body (TB) fibers in cats were presented.

#### 2.2.4. Data Length ***D*** and SAC Bin Width **ω** Analysis

To test the effect of the data length *D* on the SAC and CI, we simulated 2,000 Poissonian spike trains of one unit responding to a 100 ms long 500 Hz pure tone stimulus with a temporal resolution of 2 μs. The target VS value was 0.8 and the mean firing rate $\bar{\lambda }$ was set at 200 spikes/s. We calculated the SAC for spikes in the time interval between 30 and 80 ms after the stimulus onset with the bin width ω = 50 μs, such that ω/dt = 25. The simulation results were then compared with our theoretical findings in Equation (6).

To evaluate the effect of the SAC bin width ω, we created 400,000 trials of inhomogeneous Poissonian spike trains, each of which was 100 ms long, locked at 500 Hz with an average rate of 200 spikes/s and a target VS of 0.6. To calculate the mean and standard deviation of SAC and CI, we divided them into 1000 repetitions, each containing 400 trials. The temporal resolution of the spike trains was dt = 2μs. In total, 88 different bin width values ranging from ω= 2 to 2,000 μs were used. As shown in Results (section 3.4), deviation from the theoretical value critically depends on the value of ω/dt. Therefore, we divided the values of ω into the four groups “odd” (N = 23), “even” (N = 23), “non-integer” (N = 32), and “large” (N = 10), according to the value of the ratio ω/dt. The group “large” contains all bin widths with ω/dt≥550. For each repetition and each bin width ω, SAC and CI were computed and compared with the theoretical CI decay in Equation (7).

## 3. Results

The main goal of the present study is to establish a clear relationship between VS and CI. After a brief mathematical description of their relationship, we test the validity of our theoretical formulation with published auditory neuron models and physiologically recorded *in vivo* data. Finally, we investigate potential causes for deviations between theoretical and empirical results.

### 3.1. Mathematical Formulation of the Relation Between VS and CI

In order to relate VS and CI by mathematical expressions, we assumed that phase-locked spike times can be described by the arrival times of an inhomogeneous Poisson process with a periodic, time-dependent intensity function λ(*t*) that was based on the *von Mises* distribution (Equation 2). This function captures the periodic spike rate change at frequency *f*, the degree of phase-locking quantified with the concentration parameter κ, and the mean discharge rate $\bar{\lambda }$ of the neuronal response. A large value of κ generally corresponds to prominent phase-locking, which is indicated in the vertical alignment of spikes in raster plots (compare Figures 2A1–C1). Temporal fidelity and reproducibility of spike timings can be visualized with phase histograms (Figures 2A2–C2) or SACs (Figures 2A3–C3).

As summarized in Materials and Methods and detailed in Supplementary Material, we obtained the formulae VS(κ) = *I*_1_(κ)/*I*_0_(κ) (Equation 3) and $\text{CI}\left(\kappa \right)=I_{0}\left(2\kappa \right)/I_{0}{\left(\kappa \right)}^{2}$ (Equation 5). Here, both metrics depend only on the concentration parameter κ of the *von Mises* distribution (Figures 2D,E). Since both VS and CI are monotonic functions of κ, we can connect VS and CI using their one-to-one relationship with κ (Figure 2F). CI is a monotonically increasing function of VS (and vice versa), and VS = 0 corresponds to CI = 1. Next, we compare these results to simulated and *in vivo* data.

### 3.2. Validation of Theoretical Results

#### 3.2.1. Simulated Data

**Figure 3:**
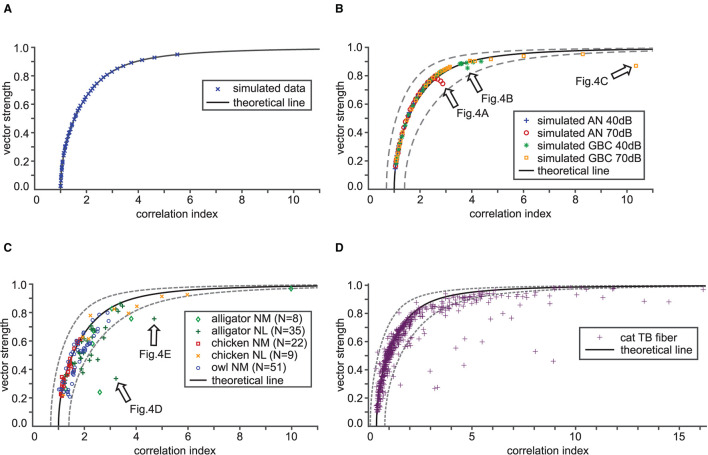
Validation of the theoretical relation between VS and CI. In each panel, the theoretical VS-CI relationship is shown with a black solid curve, while simulated or recorded data are shown by different types of symbols. The gray dotted lines indicate 0.7 × (theoretical CI) and 1.4 × (theoretical CI) for each value of VS. **(A)** VS and CI values for numerically calculated *von Mises* distributions. Results from 46 simulated units that are locked at 500 Hz with target VS values ranging from 0.05 to 0.95 (in steps of 0.02) are shown. **(B)** Simulated auditory nerve (AN) and globular bushy cell (GBC) responses with 58 units each. The stimulus frequency was fixed to the unit's CF that ranged from 200 to 2,000 Hz, each tested at 40 and 70 dB SPL. **(C)** VS-CI relationship from 125 auditory brainstem neurons of three animal species. **(D)** VS-CI relationship in 564 tonal responses of cat TB fibers. Data points were adapted from Joris et al. (2006) with permission. Arrows in **(B,C)** indicate the examples used in Figure 4.

In order to validate the theoretical relationship between VS and CI, we simulated spike trains for varied degrees of phase-locking (three examples are shown in Figures 2A1–C3) using an inhomogeneous Poisson process as described in the Materials and Methods section. From these sequences, we computed VS and CI values, and compared them with the theoretical prediction shown in Figure 2F. When the underlying spiking distribution is *von Mises*, the theoretical findings match the empirical calculations of simulated data (Figure 3A). Hence, the validity of our analytical results is confirmed with sufficiently long trials containing sufficiently many spikes.

#### 3.2.2. AN/GBC Model Data

We generated spike data using AN and GBC models driven by tonal stimulation of frequencies from 200 to 2,000 Hz at 40 and 70 dB SPL and determined VS and CI of these simulated traces. Overall, their trend closely follows the mathematical prediction (Figure 3B). There are a few instances, however, that notably deviated from the theoretical curve. These instances indicate either higher CI or lower VS than what is estimated from the theoretical relationship (e.g., arrows in Figure 3B). We will examine this discrepancy in section 3.3.

#### 3.2.3. *In vivo* Data

Finally, we analyzed auditory brainstem neuron data from three different species and two different neuron types. The animals were presented with pure tone stimuli at the unit's presumed CF. The empirical data points generally followed the trend of the theoretical curve (Figure 3C). However, considerable scattering of the VS-CI points around the theoretical prediction is evident. Most of the deviating data points appear to the right of the theoretical line, indicating a degraded VS, an increased CI, or both. The same holds true for cat TB data originally collected by Joris et al. (2006) (Figure 3D). While most data points fall into a reasonable range around the mathematical reference, there are some units with potentially reduced VS or increased CI. In summary, both simulated and empirical data were consistent with our theoretical relations between VS and CI. Possible reasons for the deviation include violations of the mathematical assumptions and systematic calculation errors of the empirical VS and CI values, which we further investigate in the following two subsections.

### 3.3. Violation of the Mathematical Assumptions

**Figure 4:**
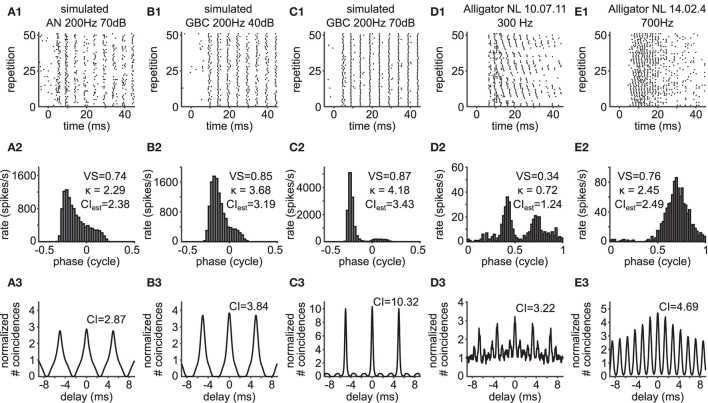
Violation of the theoretical assumptions for the VS-CI relationship. Each column shows the response of a unit whose CI value deviates from the theoretical value estimated from its VS. Corresponding data points are indicated by arrows in Figures 3B,C. **(A1–E1)** Raster plots. The sound stimulus starts at time zero. **(A2–E2)** Phase histograms with VS values, from which the value of κ was back-calculated and then the value of CI was estimated. **(A3–E3)** SACs with CI values. Note the difference between the estimated CI in **(A2–E2)** and actual CI in **(A3–E3)**. To the alligator NL units, pure tone stimuli were presented binaurally with an interaural time difference ranging from –2,000 to +2,000 μs in a step of 250 μs, repeated three times **(D1–D3)**; or from –1,000 to +1,000 μs in a step of 100 μs, repeated 5 times **(E1–E3)**; only 51 repetitions are shown in **(E1)**, but all repetitions are used for **(E2,E3)**.

Deviation from the theoretical relationship between VS and CI can happen, when the underlying spiking pattern does not obey the *von Mises* distribution function. Example rasters are shown in Figures 4A1–E1. The phase histograms of such units include skewed (Figures 4A2,B2) or bimodal (Figures 4C2,D2) phase distributions. Similar phase histograms were also observed in, e.g., cat AN fiber recordings, primarily due to harmonic distortions originating from the auditory periphery (see Peterson and Heil, 2019, and references therein). In one of the alligator NL neurons (Figure 4D1), both the spike rate and the spike timing depend on interaural time differences, leading to a bimodal phase histogram (Figure 4D2).

Such violations of the theoretical assumption of a symmetrical, unimodal *von Mises* distribution result in SACs that are also skewed (Figures 4A3,B3) or with multiple side peaks (Figures 4C3,D3). Consistent with the observations by Joris et al. (2006), we find that units with these types of phase histograms show a reduced VS. The CI, on the other hand, seems more robust to such changes, as the SAC can capture temporal structures that are not frequency specific (Parida et al., 2021). In other words, CI can be large when there is trial-to-trial reproducibility in the spiking responses even without periodic patterns. In the cases presented here, low frequency units (typically below 500 Hz) predominantly showed such phase histograms, which often present more than one peak in one stimulus cycle (i.e., peak splitting).

**Figure 5:**
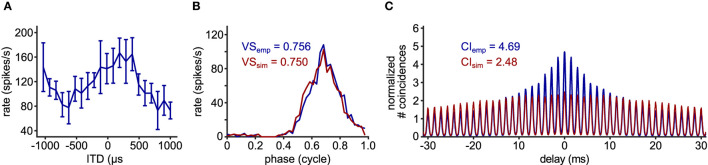
Comparison of empirical and simulated data. **(A)** ITD-tuning curve of the 700 Hz alligator NL unit used for Figures 4E1–E3. Error bars represent standard deviations (5 repetitions for each ITD). **(B)** Phase histograms and **(C)** SACs of the same NL unit (blue) and simulated spike train (red) that was generated with an inhomogeneous Poisson process whose intensity function is modeled with the empirical phase histogram without any additionally assumed trial-to-trial relations.

In addition to these relatively apparent examples, the value of CI can be higher than the theoretical prediction, when there is some hidden temporal structure between trials. Such an example is shown in Figure 4E1. In this case, the neuron was stimulated binaurally with a time difference between the two ears that was systematically varied across trials. The spike rate of this neuron varies according to the interaural time difference (ITD: Figure 5A), which is a representative response of an NL neuron (Carr et al., 2009). In contrast to the previous example (Figures 4D1,D2), the stimulus-dependent variation of spiking pattern in this neuron is hardly noticeable in the phase histogram (Figure 4E2). The CI value of this neuron, however, was substantially higher than the naive expectation from the VS (Figure 4E3). In order to investigate how such a large CI value was achieved, we generated artificial spike trains with the same phase histogram as this neuron (Figure 5B). In contrast to the ITD dependence of the actual NL neuron (Figures 4E1, 5A), the simulated spike trains contained no additional structures in their repeated trials. For delays between –10 and +10 ms, the empirical SAC (Figure 5C, blue) presents higher peaks than the simulated SAC (Figure 5C, red), indicating that spikes of this alligator NL unit were more likely to occur at the same or neighboring cycles than an inhomogeneous Poisson process having the same phase histogram. For larger delays, however, empirical spike coincidences drop below the simulated data. In sum, the deviation of the empirical CI from the theoretically expected value suggested an additional temporal structure in the spike trains of this NL unit and the comparison of empirical and simulated SACs revealed increased spike coincidences across trials.

### 3.4. Data Length and Bin Width Analysis for the SAC and CI

**Figure 6:**
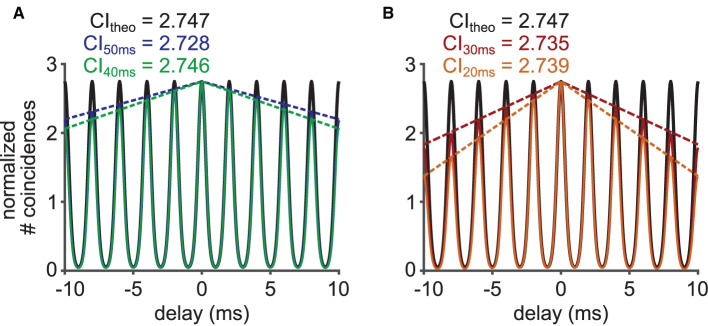
Effects of limited data length on the heights of the side peaks of the SAC. **(A)** (Black) Theoretical SAC for a spike train of an infinite length for a 500 Hz stimulation. (Blue) SAC for a 50 ms long interval of a simulated spike train with theoretical decay of SAC peak heights (dotted blue). (Green) SAC for a 40 ms long interval of a simulated spike train with theoretical decay of SAC peak heights (dotted green). The concentration parameter κ was 2.8713 corresponding to a VS of 0.8. **(B)** More examples for different data lengths of 30 (red) and 20 (orange) ms.

The choice of parameters for CI calculation can lead to an additional deviation from the theoretical prediction. We investigated the effects of the data length *D* and the SAC bin width ω on CI by deriving Equations 6 and 7. The limited data length *D* of spike trains linearly scales down the heights of the side peaks in the SAC (Figure 6A) with a multiplicative factor 1−|*s*/*D*|. This decay of SAC reflects the fact that spike coincidences are not counted for spikes that could occur before the beginning or after the end of the time interval used for the calculation of SAC. Therefore, the decay of SAC is stronger for smaller *D* (Figure 6B). The value of CI, or the SAC peak at *s* = 0, is nevertheless unaffected by the data length *D*. We also note that the value of VS is generally unaffected by *D*, as long as a sufficient number of spikes is collected.

**Figure 7:**
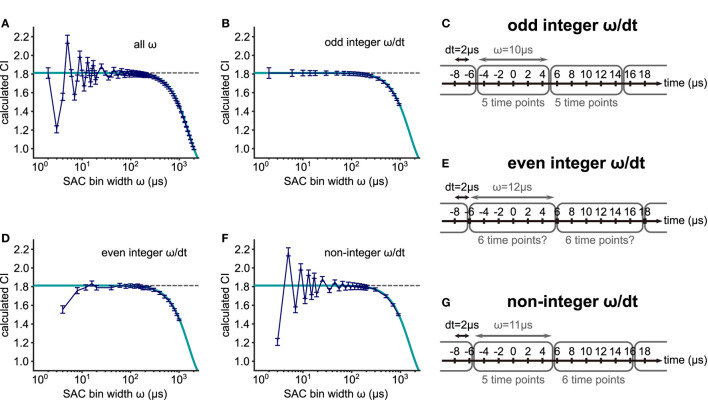
Effects of the SAC bin width ω on CI. **(A)** CI values for 88 different SAC bin widths. Spike trains simulated with a *von Mises* distribution (frequency = 500 Hz; concentration κ = 1.5157; target VS = 0.60; mean spike rate = 200 spikes/s; time step dt = 0.002 ms; duration = 100 ms; and 400 repetitions) were used. The amount of error or bias in CI depends on the ratio between the spike sampling time step dt and the SAC bin width ω, namely ω/dt, being either **(B)** odd integer, **(D)** even integer or **(F)** non-integer. The gray dotted line represents the estimated CI (= 1.8120) based on the underlying κ value. The dark blue line shows the mean CI values with error bars indicating the standard deviation for the simulated spike trains. The theoretical decay of CI with large ω is illustrated by the cyan curve (see Equation 7). **(C,E,G)** Schematic illustrations for the effects of the SAC bin width ω. **(C)** For an odd integer ratio ω/dt, each bin has the same number of time points. **(E)** When ω/dt is an even integer, the number of time points in each bin may or may not be equal depending on the rounding algorithm. **(G)** For a non-integer ω/dt, the number of time points differs between two neighboring bins.

The calculation of VS is affected by the recording time resolution (Ashida and Carr, 2010). Similarly, CI is impacted by the SAC bin width ω. For experimental data of cat AN and TB fibers, Joris et al. (2006) had already shown a decay of CI with increasing bin widths ω, which was theoretically confirmed in our calculation (Equation 7). The true CI is underestimated for large ω and decays toward 1 for ω≫100 μs for this 500 Hz example (Figure 7A), because a large ω effectively works as a low-pass filter by averaging out the spike rate in each bin. Since the decay factor in Equation (7) is a function of the product ω*f*, the decay of CI starts at different values of ω, depending on the frequency *f*. The decaying part of the curve in Figure 7A will shift to the left (smaller ω) if the frequency is increased. This frequency dependence is further investigated below.

Using a small bin width ω, however, is not always optimal for error reduction. If ω is too small, the SAC becomes more variable leading to strong deviations from the theoretical CI value (ω <50 μs in Figure 7A). For small ω, three different cases should be considered, depending on the value of the ratio between the SAC time bin and spike timing resolution dt. When the value of ω/dt is an odd integer, the simulated data closely follow the theoretical value (Figure 7B). In this case, the number of data points in each SAC bin is constant (Figure 7C). When the value of ω/dt is an even integer, however, the calculation of CI can be erroneous (Figure 7D), because the time points that are located on the border of two neighboring SAC time bins are pushed into one of the bins possibly in an uneven way, depending on the rounding algorithm used in the specific analysis program (Figure 7E). We note that this rounding issue might appear differently, if the CI bin were not centered at 0 μs. Finally, if the quotient ω/dt is not an integer, the error can be large (Figure 7F), because the number of time points can vary between SAC bins (Figure 7G).

**Figure 8:**
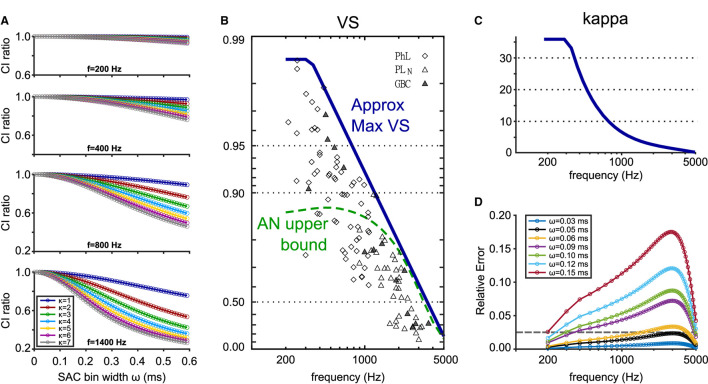
Estimation of the binning error in CI calculation depending on the bin size ω, frequency *f*, and concentration parameter κ. **(A)** Theoretical CI decay (see Equation 7) for four different frequencies and seven different κ values. CI ratio was calculated as CI_ω_/CI. **(B)** Empirical VS values measured in cats and their maximum values approximated by a function ${\text{VS}}_{\text{max}}=\min\left\{0.986,1-{\left(f/5700\right)}^{1.5}\right\}$. Symbols show cat TB fiber recordings (Joris et al., 1994). Diamonds: phase-locked units; open triangles: presumed GBCs with primary-like-with-notch PSTHs; filled triangles: histologically confirmed GBCs. Dashed green line indicates the maximal VS values for cat AN fibers (Johnson, 1980). Figure adapted from Ashida et al. (2019). **(C)** Estimation of κ_max_ from VS_max_ in **(B)** calculated with Equation (3). **(D)** Upper CI error bounds for different bin widths calculated with the estimated κ_max_ shown in **(C)**. Relative errors were computed as (CI−CI_ω_)/CI. The gray dotted horizontal line highlights the relative error of 2.5 %.

Based on these theoretical considerations, we aim to derive a recommendation for the choice of reasonable SAC bin widths. In addition to ω, the error in CI depends both on the frequency *f* and the concentration parameter κ that controls the degree of phase-locking (Equation 7). For a given frequency, more prominent phase-locking, reflected in large κ values, leads to a more pronounced CI decay (Figure 8A) because the strength of the n-th harmonic (see Equation 7) monotonically increases with κ. This observation allows us to estimate upper CI error bounds by only considering the maximal value of κ that is physiologically plausible at each frequency. Using published data in cats (Joris et al., 1994), we obtained an empirical approximation for the maximum values of VS at each frequency (Figure 8B, blue line) and calculated the corresponding κ_max_ (Figure 8C). The values of κ_max_ for AN fibers also matched the estimation by Peterson and Heil (2019, see their Figure 9). Our results suggest that a bin width of ω = 50 μs keeps the relative error below 2.5 % for frequencies in the range of 200 and 5,000 Hz (Figure 8D). The error is largest for frequencies between 2,000 and 3,000 Hz.

In summary, the SAC bin width ω should be chosen to be equal to or smaller than 50 μs to avoid a substantial underestimation of CI. Furthermore, the amount of error in CI calculation depends on the ratio between the bin width ω and the spike sampling time step dt. The bias is smallest in the odd integer case (Figures 7B,C) and highest in the non-integer case (Figures 7F,G).

## 4. Discussion

In the era of large-scale recording and simulation, the ability to compare data has become a fundamental issue in various scientific fields, including biology (Cao et al., 2005; Vogt, 2009; Mallott et al., 2019) and neuroscience (Mulugeta et al., 2018; Bzdok et al., 2019). A number of practical suggestions have been made and implemented toward effective integration of multiple datasets (e.g., Poldrack and Gorgolewski, 2014; Zehl et al., 2016; Tratwal et al., 2020). These ongoing efforts, however, do not necessarily guarantee that neurophysiological data that were published decades ago can be compared with more recent data quantified with different methods. As reviewed in the Introduction, VS has served as a practical measure of phase-locking, since it was introduced more than half a century ago (Goldberg and Brown, 1969). More recently, the use of CI has been increasingly common, since SACs and CIs can be defined for a wider variety of stimuli than VS, including non-periodic acoustic stimulation (Joris et al., 2006). To facilitate comparisons between phase-locked spiking data quantified with VS and those with CI, the present study established a mathematical relationship between these measures (Figure 2F). Our mathematical formulation assumed a *von Mises* distribution characterized with the concentration parameter κ that systematically affects both VS and CI (Figures 2D,E).

Our theoretical predictions generally agreed with measured (Figures 3C,D) or simulated (Figures 3A,B) spiking data of several different auditory neurons, even though spike timings in real neurons do not necessarily follow the *von Mises* distribution (Peterson and Heil, 2019). Real neurons and modeled AN fibers, for example, have refractory periods, while *von Mises* distributed spike trains do not. Recordings of auditory nerve fibers showed that refractoriness can affect VS especially at low frequencies (below 800 Hz; Avissar et al., 2013; Peterson and Heil, 2019). Furthermore, spiking timing of auditory nerve fibers and cochlear nucleus neurons are generally more regular than pure Poissonian spike trains (Rothman et al., 1993; Heil and Peterson, 2017). The AN and GBC models we used (Bruce et al., 2018; Ashida et al., 2019) include refractory periods and show more regular spiking than the Poisson process. Nevertheless, our simulation results with these models showed a good agreement with the curve for the *von Mises* distribution (Figure 3B), suggesting that the violations of these mathematical assumptions may only have limited effects on the theoretical VS-CI relationship. More systematic analyses on the effects of refractoriness and regularity on SAC would be a topic of future studies.

Deviations of data points from the theoretical curve may originate from a skewed (Figures 4A2,B2) or bimodal (Figures 4C2,D2) phase distribution (Peterson and Heil, 2019). In auditory nerve recordings *in vivo*, such bimodal distributions were observed, for example, with low-frequency, high-intensity acoustic stimulation (Johnson, 1980; Wei et al., 2017) which induces considerable harmonic distortions in the peripheral response (Peterson and Heil, 2019, also see references therein for more examples). In addition to these relatively apparent cases, the value of CI can be higher than what is expected from VS, even if the phase distribution resembles a *von Mises* distribution. In our example (Figure 4E2), spikes across trials were more likely to occur in the same or neighboring stimulus cycles than naively expected from the period histogram that only retains the phase information of spikes in each period (Figures 5B,C). These results indicate that the applicability of our theoretical results is limited to unimodal (*von-Mises*-like) phase distributions without additional trial-to-trial coincidences due to variations of the stimulus parameters. A more appropriate fitting of skewed or bimodal phase distribution would require modifications of the fitting function by, e.g., summing multiple unimodal distributions or introducing additional shape parameters to create asymmetry (Gatto and Jammalamadaka, 2007; Umbach and Jammalamadaka, 2009; Abe and Pewsey, 2011; Kim and SenGupta, 2013). In the cat auditory nerve, for example, additional one or two distortion components would be necessary to account for the observed spiking patterns (Peterson and Heil, 2019).

In addition to the violation of theoretical assumptions, multiple sources of errors can affect the calculation of CI. While the length *D* of each trial would not affect the value of CI (Figure 6), the size of the SAC time bin ω can constitute a major error source (Figure 7). If ω is too large, the resulting CI value becomes smaller than the theoretical estimation, because the SAC peak is reduced by averaging within the time bin. A similar effect was shown for the calculation of VS (Ashida and Carr, 2010). For small ω, the calculation of CI can be error-prone, especially when the number of data points is not uniform across SAC bins (Figures 7D,F). In other words, the size of SAC bins should be selected in a way that the number of time steps in each bin becomes constant (Figures 7B,C). Considering these factors, we support the selection of ω = 50 μs (or some similar value), which was used by the original study of Joris et al. (2006), provided that the recording time step is sufficiently small (e.g., 10 μs or less). In our *in vivo* recording data, however, the spike times were usually sampled at a lower rate (48 kHz, corresponding to a 20.8 μs time step) due to technical restrictions. Hence, the discrepancy between the theoretical prediction and empirical data (Figure 3C) could be (at least partly) due to the effect of the SAC bin size. Furthermore, the number of repeated trials needs to be large enough to have sufficiently many spike coincidences in each SAC time bin. Otherwise, the empirical CI value may differ from the theoretical prediction, which is exemplified by larger error bars for small ω (Figure 7B). Moreover, for an estimation of CI from VS, even a small error in VS may result in a large error of CI, especially for VS>0.95. This is because the value of VS is bounded by one, while the value of CI can be infinitely large (Figure 2F).

Comparisons of multiple measures may reveal hidden structures of spike trains. As demonstrated in Figures 5B,C, a comparison of the VS-CI relationship with the theoretical prediction may help us discover additional spiking patterns that may not be found with a period histogram or SAC alone. While CI contains broadband spectral information, VS is a measure at a specific frequency (Parida et al., 2021). Expanding the concept of VS is possible by introducing multiple reference frequencies (van Hemmen and Vollmayr, 2013). For spectrally rich acoustic stimuli, such as natural sounds, locking to more than one frequencies may have to be considered (Joris et al., 2004). Even though the use of natural stimuli is increasing common in neuroscience, response patterns to simple stimuli still provide fundamental information about the functionality of the sensory system (e.g., Rust and Movshon, 2005). It is not always clear, however, how neuronal responses to simple stimuli can be related with those to complex stimuli. Future investigation on the relationship between CI and frequency-dependent VS under physiologically reasonable mathematical conditions might be useful to bridge between fundamental spiking patterns of auditory neurons and responses to broadband acoustic stimulation.

A number of measures have been developed to quantify the degree of correlation between spike trains, including SACs and its variations (reviewed in Cutts and Eglen, 2014). Nevertheless, quantitative relationships between these measures are not always clear. Gai and Carney (2008) compared CI, the Victor-Purpura spike distance metric, and mutual information to quantify time-locked spiking patterns recorded in the cochlear nucleus. Our theoretical method used in this study may also be applied to temporal measures other than VS and CI. Such an analysis would not only reveal the mathematical relations behind these measures but also provide practical information on which measure to use for quantifying temporal information processing in the nervous system.

## Data Availability Statement

The original contributions presented in the study are included in the article/Supplementary Material, further inquiries can be directed to the corresponding author. Matlab program code for some of the simulations and analyses in this study is available online at https://github.com/pinkbox-models/CI-VS-2021.

## Ethics Statement

Ethical review and approval was not required for the animal study because only existing data collected in previously published studies were re-analyzed.

## Author Contributions

DK and GA: conceptualization, formal analysis, numerical simulation, data analysis, interpretation, visualization, and drafting manuscript. CC and GA: physiological data collection. CC and JK: resources. CC, JK, and GA: funding acquisition. JK and GA: project administration and supervision. DK, CC, JK, and GA: reviewing and editing manuscript and approving submission. All authors contributed to the article and approved the submitted version.

## Funding

This work was supported by the Deutsche Forschungsgemeinschaft (DFG, German Research Foundation) under Germany's Excellence Strategy EXC2177/1 (Project ID 390895286), Associate Junior Fellowship from the Hanse-Wissenschaftskolleg (GA), and NIH/NIDCD Grant R01DC019341 (CC).

## Conflict of Interest

The authors declare that the research was conducted in the absence of any commercial or financial relationships that could be construed as a potential conflict of interest.

## Publisher's Note

All claims expressed in this article are solely those of the authors and do not necessarily represent those of their affiliated organizations, or those of the publisher, the editors and the reviewers. Any product that may be evaluated in this article, or claim that may be made by its manufacturer, is not guaranteed or endorsed by the publisher.
