# Supplementary material for: Theoretical Relationship Between Two Measures of Spike Synchrony: Correlation Index and Vector Strength
Source: Front Neurosci. 2021 Dec 20;15:761826. doi: 10.3389/fnins.2021.761826 (PMC8721039; doi:10.3389/fnins.2021.761826)
Supplement: Supplementary file 1 [file Data_Sheet_1.pdf]

Supplementary Material

In this supplementary material, we provide detailed derivations of the mathematical formulae used in our main article (Kessler et al., 2021).

1 DERIVATION OF SAC AND CI

For the entire section, we assume that the reference frequency f is known and given.

Here is the definition of the 2π-periodic, normalized *von Mises* density function with mean phase $\mu = 0$ for frequency f on the radiant domain, $x \in [0, 2\pi]$:

$$p(x \mid \kappa) = \frac{1}{2\pi \cdot I_0(\kappa)} \cdot \exp\big(\kappa \cos(x)\big), \quad 0 \leq \kappa < \infty, \tag{S1}$$

with κ being the concentration parameter, and $I_0(\kappa)$ the modified Bessel function of the first kind and order zero:

$$I_0(\kappa) = \frac{1}{2\pi} \int_0^{2\pi} \exp\big(\kappa \cos(\psi)\big) \mathrm{d}\psi\,.$$

The equivalent formulation for the T-periodic, normalized *von Mises* density on the real domain $\mathbb{R}_{[0,T]}$ is

$$p(t \mid \kappa) = \frac{1}{T \cdot I_0(\kappa)} \cdot \exp\big(\kappa \cos(2\pi f t)\big), \quad 0 \leq \kappa < \infty, \tag{S2}$$

with $T = 1/f$ being the period length.

The continuous extension of the definition of vector strength (VS) is given as

$$\text{VS} = \frac{1}{T} \left| \int_0^T p(t) \cdot \exp(2\pi i f t)\, \mathrm{d}t \right|, \tag{S3}$$

where $p(t)$ is the underlying T-periodic density function.

Assuming a *von Mises* distribution $p(t \mid \kappa)$ from above in Eq. (S2), the relation between VS and the concentration parameter κ is given as

$$\begin{aligned}
\text{VS}(\kappa) &= \left| \int_0^T \frac{1}{T\, I_0(\kappa)} \cdot \exp\big(\kappa \cos(2\pi f t)\big) \cdot \exp(2\pi i f t)\, \mathrm{d}t \right| \\
&= \frac{1}{I_0(\kappa)} \left| \frac{1}{T} \int_0^T \exp\big(\kappa \cos(2\pi f t)\big) \cdot \big(\cos(2\pi f t) + i \cdot \sin(2\pi f t)\big)\, \mathrm{d}t \right| \\
&= \frac{1}{I_0(\kappa)} \cdot \left| \frac{1}{T} \int_0^T \exp\big(\kappa \cos(2\pi f t)\big) \cdot \cos(2\pi f t)\, \mathrm{d}t \right| \\
&= \frac{I_1(\kappa)}{I_0(\kappa)}\,,
\end{aligned}$$

where $I_1(\kappa)$ is the modified Bessel function of the first kind and order one:

$$I_1(\kappa) = \frac{1}{2\pi} \int_0^{2\pi} \exp(\kappa \cos(\psi))\; \cos(\psi)\, \mathrm{d}\psi\,.$$

Now, we derive an expression for the SAC. Let $\varphi_{X_1}, \varphi_{X_2}$ be two T-periodic normalized density functions. For the general case, when $X_1 \sim \varphi_{X_1}$ and $X_2 \sim \varphi_{X_2}$ are stochastically independent (i.e., the firing of all periods within a trial is pairwise independent and identical and so is the firing between different trials), we can convolve their density functions with each other to find the density function φ_S of $S = X_2 - X_1$:

$$\varphi_S(s) = (\varphi_{X_2} * \varphi_{-X_1})(s) = \int_0^T \varphi_{X_2}(t) \cdot \varphi_{-X_1}(s-t)\mathrm{d}t\,.$$

For our case, when X_1 and X_2 are identically *von Mises* distributed, it holds that $\varphi_{X_2}(t) = \varphi_{X_1}(t) = p(t \,|\, \kappa)$. Since the *von Mises* distribution is symmetric, $\varphi_{-X_1}(s-t) = \varphi_{X_1}(t-s)$, and we get:

$$\begin{aligned}
\varphi_S(s \,|\, \kappa) &= \int_0^T p(t \,|\, \kappa) \cdot p(t-s \,|\, \kappa)\mathrm{d}t \\
&= \int_0^T \frac{1}{T \cdot I_0(\kappa)} \exp(\kappa \cos(2\pi f t)) \cdot \frac{1}{T \cdot I_0(\kappa)} \exp(\kappa \cos(2\pi f (t-s)))\,\mathrm{d}t \\
&= \frac{1}{T^2 \cdot I_0(\kappa)^2} \int_0^T \exp(\kappa(\cos(2\pi f t) + \cos(2\pi f (t-s)))\,\mathrm{d}t \\
&= \frac{1}{T \cdot I_0(\kappa)^2} \cdot \frac{1}{T} \int_0^T \exp\left(2\kappa \cos(\pi f s) \cdot \cos\left(2\pi f \left(t - \frac{s}{2}\right)\right)\right) \mathrm{d}t \\
&= \frac{I_0(2\kappa \cos(\pi f s))}{T \cdot I_0(\kappa)^2}\,.
\end{aligned}$$

In the third step, we applied the formula $\cos(a) + \cos(b) = 2 \cdot \cos(\frac{a+b}{2}) \cdot \cos(\frac{a-b}{2})$ with $a = 2\pi f t$ and $b = 2\pi f(t-s)$. For the last step, we used the definition of the modified Bessel function of first kind, since $\cos(2\pi f(t - \frac{s}{2}))$ is T-periodic, and $2\kappa\cos(\pi f s)$ is a scalar (independent from the integration variable t). The resulting expression tells us how probable the delays between spikes are. In order to derive the SAC from this, we need to find a scaling constant c, such that the mean function value over $[0, T]$ is one:

$$\frac{1}{T-0} \int_0^T c \cdot \varphi_S(s \,|\, \kappa)\,\mathrm{d}s \overset{!}{=} 1\,.$$

Because φ_S is a normalized probability density over $[0, T]$ by definition, we find $c = T$. Then, the final formula for SAC looks like the following:

$$\text{SAC}_\kappa(s) = T \cdot \varphi_S(s) = \frac{I_0(2\kappa \cos(\pi f s))}{I_0(\kappa)^2}\,. \tag{S4}$$

Now, we can simply calculate the correlation index as a function of κ by evaluating the SAC from Eq. (S4) at delay $s = 0$:

$$\text{CI}(\kappa) = \text{SAC}_\kappa(0) = \frac{I_0(2\kappa\cos(\pi f \cdot 0))}{I_0(\kappa)^2} = \frac{I_0(2\kappa)}{I_0(\kappa)^2}\,. \tag{S5}$$

2 EFFECT OF DATA LENGTH AND SAC BIN WIDTH

2.1 Effects of trial length D on SAC and CI.

Let X be a uniform random variable on $[0, D)$. The probability that $X + s$ for an arbitrary delay s is still within the interval $[0, D)$ is given as

$$\begin{aligned}
\zeta_D(s) &= \mathbb{P}\big(X + s \in [0, D) \,\big|\, X \in [0, D)\big) \\
&= \mathbb{P}(-s \le X < D - s \,\big|\, 0 \le X < D) \\
&= \begin{cases} \mathbb{P}(0 \le X < D - s \,\big|\, 0 \le X < D), & 0 \le s < D \\ \mathbb{P}(-s \le X < D \,\big|\, 0 \le X < D), & -D < s \le 0 \\ 0\,, & |s| \ge D \end{cases} \\
&= \begin{cases} 1 - \frac{s}{D}, & 0 \le s < D \\ 1 + \frac{s}{D}, & -D < s \le 0 \\ 0\,, & |s| \ge D \end{cases} \\
&= \begin{cases} 1 - \left|\frac{s}{D}\right|, & |s| < D \\ 0\,, & |s| \ge D \end{cases}\,.
\end{aligned}$$

Assuming that X is stochastically independent from the random variable describing the number of normalized coincidences captured in the SAC, their joint distribution is given as the product of their marginal distributions. Hence, the above triangle function serves as a multiplicative factor for the theoretical SAC derived in Equation (S4) to capture the effect of data length D. The CI remains unaffected by D because the multiplicative factor at $s = 0$ is just $\zeta_D(0) = 1$.

$$\begin{aligned}
\text{SAC}_{\kappa,D}(s) &= \text{SAC}_\kappa(s) \cdot \zeta_D(s) \\
&= \begin{cases} \frac{I_0(2\kappa\cos(\pi f s))}{I_0(\kappa)^2}\left(1 - \left|\frac{s}{D}\right|\right), & |s| < D \\ 0\,, & |s| \ge D \end{cases}\,.
\end{aligned}$$

2.2 Effects of (large) SAC bin width ω on SAC and CI.

We take the *von Mises* distribution $p(t \,|\, \kappa)$ on the real domain, as defined in Eq. (S2):

$$p(t \,|\, \kappa) = \frac{1}{T \cdot I_0(\kappa)}\, \exp\big(\kappa\, \cos(2\pi f t)\big)\,. \tag{S6}$$

Its Fourier series is given as

$$p(t \,|\, \kappa) = \frac{1}{T}\left(1 + 2\sum_{n=1}^{\infty} \frac{I_n(\kappa)}{I_0(\kappa)} \cos(2\pi n f t)\right), \tag{S7}$$

since

$$a_0 = \frac{1}{2\pi}\int_{-\pi}^{\pi} p(t\,|\,\kappa)\,\mathrm{d}t = \frac{1}{T}\,,$$
$$a_k = \frac{1}{\pi}\int_{-\pi}^{\pi} p(t\,|\,\kappa)\,\cos(kt)\,\mathrm{d}t = \frac{2}{T}\cdot\frac{I_k(\kappa)}{I_0(\kappa)}\,,\ \text{for } k = 1,2,3,\dots\,.$$

All of the sine terms $b_k = \frac{1}{\pi}\int_{-\pi}^{\pi} p(t\,|\,\kappa)\sin(kt)\,\mathrm{d}t$ vanish because they are odd functions.

We define the window function $\mathsf{w}(t)$ as

$$\mathsf{w}(t\,|\,\omega) = \begin{cases} 1/\omega, & -\omega/2 \le t \le \omega/2 \\ 0, & |t| > \omega/2 \end{cases}\,, \tag{S8}$$

and convolve it with the Fourier series from Eq. (S7):

$$\begin{aligned}
g(t\,|\,\kappa,\omega) &:= (\mathsf{w} * p)(t\,|\,\kappa) = \int_{-\infty}^{\infty} \mathsf{w}(t-\tau\,|\,\omega)\,\cdot\,p(\tau\,|\,\kappa)\,\mathrm{d}\tau \\
&= \frac{1}{\omega}\int_{t-\frac{\omega}{2}}^{t+\frac{\omega}{2}} p(\tau\,|\,\kappa)\,\mathrm{d}\tau \\
&= \frac{1}{T\omega}\int_{t-\frac{\omega}{2}}^{t+\frac{\omega}{2}} \left(1 + 2\sum_{n=1}^{\infty}\frac{I_n(\kappa)}{I_0(\kappa)}\cos(2\pi n f\tau)\right)\mathrm{d}\tau \\
&= \frac{1}{T\omega}\left(\int_{t-\frac{\omega}{2}}^{t+\frac{\omega}{2}} 1\,\mathrm{d}\tau + 2\int_{t-\frac{\omega}{2}}^{t+\frac{\omega}{2}}\sum_{n=1}^{\infty}\frac{I_n(\kappa)}{I_0(\kappa)}\cos(2\pi n f\tau)\,\mathrm{d}\tau\right) \\
&= \frac{1}{T\omega}\left(\omega + 2\sum_{n=1}^{\infty}\frac{I_n(\kappa)}{I_0(\kappa)}\cdot\frac{\sin(2\pi n f(t+\frac{\omega}{2})) - \sin(2\pi n f(t-\frac{\omega}{2}))}{2\pi n f}\right) \\
&= \frac{1}{T\omega}\left(\omega + 2\sum_{n=1}^{\infty}\left(\frac{I_n(\kappa)}{I_0(\kappa)}\right)\left(\frac{\sin(\pi n f\omega)}{\pi n f}\right)\cos(2\pi n f t)\right) \\
&= \frac{1}{T}\left(1 + 2\sum_{n=1}^{\infty}\left(\frac{I_n(\kappa)}{I_0(\kappa)}\right)\left(\frac{\sin(\pi n f\omega)}{\pi n f\omega}\right)\cos(2\pi n f t)\right)\,,
\end{aligned}$$

where in the forth step we drew the integral into the series and assessed it, and in the second last step we used that $\sin(x) - \sin(y) = 2\cos(\frac{x+y}{2})\sin(\frac{x-y}{2})$ with $x = 2\pi n f(t+\frac{\omega}{2})$ and $y = 2\pi n f(t-\frac{\omega}{2})$. Now, we multiply g from above with the *von Mises* distribution p:

$$\begin{aligned}
h(t\,|\,\kappa,\omega) &:= g(t\,|\,\kappa,\omega)\,\cdot\,p(t\,|\,\kappa) \\
&= \frac{1}{T^2}\left(1 + 2\sum_{n=1}^{\infty}\frac{I_n(\kappa)}{I_0(\kappa)}\left(\frac{\sin(\pi n f\omega)}{\pi n f\omega}\right)\cos(2\pi n f t)\right) \\
&\quad\times\left(1 + 2\sum_{n=1}^{\infty}\frac{I_n(\kappa)}{I_0(\kappa)}\cos(2\pi n f t)\right)
\end{aligned}$$

$$
\begin{aligned}
&= \frac{1}{T^2}\Bigg(1 + 2\sum_{n=1}^{\infty} \frac{I_n(\kappa)}{I_0(\kappa)}\left(1 + \frac{\sin(\pi n f \omega)}{\pi n f \omega}\right)\cos(2\pi n f t) \\
&\qquad + 4\sum_{n=1}^{\infty}\left(\frac{I_n(\kappa)}{I_0(\kappa)}\right)^2\left(\frac{\sin(\pi n f \omega)}{\pi n f \omega}\right)\cos^2(2\pi n f t) \\
&\qquad + 4\sum_{\substack{m,n=1\\ n\neq m}}\left(\frac{I_m(\kappa)\, I_n(\kappa)}{I_0(\kappa)^2}\right)\left(\frac{\sin(\pi n f \omega)}{\pi n f \omega}\right)\cos(2\pi m f t) \\
&\qquad\qquad \times \cos(2\pi n f t)\Bigg),
\end{aligned}
$$

where we used $\cos^2(x) = \frac{1+\cos(2x)}{2}$.

Now, we can derive a formula, where CI depends not only on κ, but also on the bin width ω.

$$
\begin{aligned}
\mathsf{CI}(\kappa\,|\,\omega) &= T \cdot \int_0^T h(t\,|\,\kappa,\omega)\,\mathrm{d}t \\
&= \frac{1}{T}\int_0^T 1\,\mathrm{d}t \\
&\quad + \frac{2}{T}\int_0^T \sum_{n=1}^{\infty}\frac{I_n(\kappa)}{I_0(\kappa)}\left(1 + \frac{\sin(\pi n f \omega)}{\pi n f \omega}\right)\cos(2\pi n f t)\,\mathrm{d}t \\
&\quad + \frac{4}{T}\int_0^T \sum_{n=1}^{\infty}\left(\frac{I_n(\kappa)}{I_0(\kappa)}\right)^2\left(\frac{\sin(\pi n f \omega)}{\pi n f \omega}\right)\cos^2(2\pi n f t)\Bigg)\,\mathrm{d}t \\
&\quad + \frac{4}{T}\int_0^T \sum_{\substack{m,n=1\\ n\neq m}}\left(\frac{I_m(\kappa)\, I_n(\kappa)}{I_0(\kappa)^2}\right)\left(\frac{\sin(\pi n f \omega)}{\pi n f \omega}\right) \\
&\qquad\qquad \times \cos(2\pi m f t)\cos(2\pi n f t)\,\mathrm{d}t \\
&= \frac{1}{T}\int_0^T 1\,\mathrm{d}t + \frac{1}{T}\int_0^T 2\sum_{n=1}^{\infty}\left(\frac{I_n(\kappa)}{I_0(\kappa)}\right)^2\left(\frac{\sin(\pi n f \omega)}{\pi n f \omega}\right)\mathrm{d}t \\
&= \frac{1}{T}\big[\,t\,\big]_{t=0}^{t=T} + \frac{4}{T}\sum_{n=1}^{\infty}\left(\frac{I_n(\kappa)}{I_0(\kappa)}\right)^2 \frac{\sin(\pi n f \omega)}{\pi n f \omega}\int_0^T \cos^2(2\pi n f t)\,\mathrm{d}t \\
&= 1 + 2\sum_{n=1}^{\infty}\underbrace{\left(\frac{I_n(\kappa)}{I_0(\kappa)}\right)^2}_{\substack{\text{strength of}\\ \text{n-th harmonic}}}\underbrace{\left(\frac{\sin(\pi n f \omega)}{\pi n f \omega}\right)}_{\text{decay factor}},
\end{aligned}
$$

where in the second step, we used the fact that the second and forth summand of $h(t\,|\,\kappa,\omega)$ vanish. This is the case because $\int_0^T \cos(2\pi n f t)\,\mathrm{d}t = 0$ and $\int_0^T \cos(2\pi n f t)\cos(2\pi m f t)\,\mathrm{d}t = 0$ for

$m \neq n > 0$. In the last step, we used $2\cos^2(x) = 1 + \cos(2x)$ for calculating the integral

$$\int_0^T \cos^2(2\pi n f t)\,\mathrm{d}t = \int_0^T \frac{1}{2}\Big(1 + \cos(4\pi n f t)\Big)\,\mathrm{d}t$$
$$= \int_0^T \frac{1}{2}\,\mathrm{d}t = \frac{T}{2}\,.$$

Thus, we have derived that

$$\mathsf{CI}(\kappa \,|\, \omega) = 1 + 2\sum_{n=1}^{\infty}\left(\frac{I_n(\kappa)}{I_0(\kappa)}\right)^2\left(\frac{\sin(\pi n f \omega)}{\pi n f \omega}\right). \tag{S9}$$

Since $\frac{\sin(\pi n f \omega)}{\pi n f \omega} \to 1$ as $\omega \to 0$ for any natural number n, we have

$$\mathsf{CI}(\kappa \,|\, \omega = 0) := \lim_{\omega \to 0} \mathsf{CI}(\kappa \,|\, \omega) = 1 + 2\sum_{n=1}^{\infty}\left(\frac{I_n(\kappa)}{I_0(\kappa)}\right)^2.$$

This should be equal to $\mathsf{CI} = \frac{I_0(2\kappa)}{I_0(\kappa)^2}$, see Eq. (S5). An equivalent formulation is

$$I_0(2\kappa) = I_0(\kappa)^2 + 2\sum_{n=1}^{\infty} I_n(\kappa)^2\,.$$

We will now prove this. By definition, we have

$$I_0(2\kappa) = \frac{1}{\pi}\int_0^{\pi} \exp\big(2\kappa\cos(\theta)\big)\,\mathrm{d}\theta$$
$$= \frac{1}{\pi}\int_0^{\pi} \exp\big(\kappa\cos(\theta)\big)\cdot\exp\big(\kappa\cos(\theta)\big)\,\mathrm{d}\theta\,.$$

From Abramowitz and Stegun (1970), we know that

$$\exp\big(\kappa\cos(\theta)\big) = I_0(\kappa) + 2\sum_{n=1}^{\infty} I_n(\kappa)\cos(n\theta)\,. \tag{S10}$$

With Eq. (S10), we get a binomial formula in the expression above. After expansion of this product and using the linearity of integration, we get:

$$I_0(2\kappa) = \frac{1}{\pi}\int_0^{\pi} I_0(\kappa)^2\,\mathrm{d}\theta$$
$$+ \frac{1}{\pi}\int_0^{\pi} 4\,I_0(\kappa)\sum_{n=1}^{\infty} I_n(\kappa)\cos(n\theta)\,\mathrm{d}\theta$$
$$+ \frac{1}{\pi}\int_0^{\pi}\left(2\sum_{n=1}^{\infty} I_n(\kappa)\cos(n\theta)\right)^2\mathrm{d}\theta\,.$$

For the sake of clarity, we have a separate look at each of these three summands A1-A3.

$$\text{A1} = \frac{1}{\pi}\int_0^{\pi} I_0(\kappa)^2 \,\mathrm{d}\theta = \left[\frac{1}{\pi} I_0(\kappa)^2\,\theta\right]_{\theta=0}^{\theta=\pi} = I_0(\kappa)^2\,,$$

$$\begin{aligned}
\text{A2} &= \frac{1}{\pi}\int_0^{\pi} 4\,I_0(\kappa)\,\sum_{n=1}^{\infty} I_n(\kappa)\cos(n\theta)\,\mathrm{d}\theta = \frac{1}{\pi}\,4\,I_0(\kappa)\,\sum_{n=1}^{\infty}\int_0^{\pi} I_n(\kappa)\cos(n\theta)\,\mathrm{d}\theta \\
&= \frac{1}{\pi}\,4\,I_0(\kappa)\,\sum_{n=1}^{\infty} I_n(\kappa)\,\frac{\sin(n\pi)-\sin(0)}{n} \\
&= 0\,,
\end{aligned}$$

$$\begin{aligned}
\text{A3} &= \frac{1}{\pi}\int_0^{\pi}\left(2\sum_{n=1}^{\infty} I_n(\kappa)\cos(n\theta)\right)^2 \mathrm{d}\theta \\
&= \frac{1}{\pi}\int_0^{\pi} 4\Bigg(\sum_{n=1}^{\infty} I_n(\kappa)^2\cos^2(n\theta) \;+ \sum_{\substack{n,m=1\\ m\neq n}}^{\infty} I_n(\kappa)I_m(\kappa)\cos(n\theta)\cos(m\theta)\Bigg)\,\mathrm{d}\theta \\
&= \frac{4}{\pi}\Bigg(\sum_{n=1}^{\infty}\int_0^{\pi} I_n(\kappa)^2\cos^2(n\theta)\,\mathrm{d}\theta \;+ \sum_{\substack{n,m=1\\ m\neq n}}^{\infty}\int_0^{\pi} I_n(\kappa)I_m(\kappa)\cos(n\theta)\cos(m\theta)\,\mathrm{d}\theta\Bigg) \\
&= \frac{4}{\pi}\sum_{n=1}^{\infty}\frac{2\pi n+\sin(2\pi n)}{4n} I_n(\kappa)^2 \\
&= 2\sum_{n=1}^{\infty}\frac{\pi n+\frac{1}{2}\sin(2\pi n)}{\pi n} I_n(\kappa)^2 \\
&= 2\sum_{n=1}^{\infty} I_n(\kappa)^2\,.
\end{aligned}$$

Thus, we have proven that

$$\begin{aligned}
I_0(2\kappa) &= \text{A1}+\text{A2}+\text{A3} \\
&= \frac{1}{\pi}\int_0^{\pi} I_0(\kappa)^2\,\mathrm{d}\theta + \frac{1}{\pi}\int_0^{\pi} 4\,I_0(\kappa)\,\sum_{n=1}^{\infty} I_n(\kappa)\cos(n\theta)\,\mathrm{d}\theta + \frac{1}{\pi}\int_0^{\pi}\left(2\sum_{n=1}^{\infty} I_n(\kappa)\cos(n\theta)\right)^2\mathrm{d}\theta \\
&= I_0(\kappa)^2 + 2\sum_{n=1}^{\infty} I_n(\kappa)^2\,.
\end{aligned}$$

REFERENCES

Abramowitz, M. and Stegun, I. A. (1970). *Handbook of Mathematical Functions: With Formulas, Graphs, and Mathematical Tables* (New York: Dover Publications, Inc.)

Kessler, D., Carr, C. E., Kretzberg, J., and Ashida, G. (2021). Theoretical relationship between two measures of spike synchrony: Correlation index and vector strength. *Front Neurosci* 15,

761826. doi:10.3389/fnins.2021.761826
